# Using Machine Learning to Predict Sensorineural Hearing Loss Based on Perilymph Micro RNA Expression Profile

**DOI:** 10.1038/s41598-019-40192-7

**Published:** 2019-03-04

**Authors:** Matthew Shew, Jacob New, Helena Wichova, Devin C. Koestler, Hinrich Staecker

**Affiliations:** 1 University of Kansas School of Medicine, Department of Otolaryngology-Head and Neck Surgery, Kansas City, KS USA; 2 University of Kansas School of Medicine, Kansas City, KS USA; 3 University of Kansas School of Medicine, Department of Biostatistics, Kansas City, KS USA

## Abstract

Hearing loss (HL) is the most common neurodegenerative disease worldwide. Despite its prevalence, clinical testing does not yield a cell or molecular based identification of the underlying etiology of hearing loss making development of pharmacological or molecular treatments challenging. A key to improving the diagnosis of inner ear disorders is the development of reliable biomarkers for different inner ear diseases. Analysis of microRNAs (miRNA) in tissue and body fluid samples has gained significant momentum as a diagnostic tool for a wide variety of diseases. In previous work, we have shown that miRNA profiling in inner ear perilymph is feasible and may demonstrate distinctive miRNA expression profiles unique to different diseases. A first step in developing miRNAs as biomarkers for inner ear disease is linking patterns of miRNA expression in perilymph to clinically available metrics. Using machine learning (ML), we demonstrate we can build disease specific algorithms that predict the presence of sensorineural hearing loss using only miRNA expression profiles. This methodology not only affords the opportunity to understand what is occurring on a molecular level, but may offer an approach to diagnosing patients with active inner ear disease.

## Introduction

Hearing loss is the most common neurodegenerative disease worldwide and is estimated to affect over 432 million adults and 34 million children worldwide^1^. Unaddressed hearing loss is estimated to pose an annual global cost of over 750 billion US dollars^1^. Despite the significant disease burden and economic impact of hearing loss, diagnosing and treating this condition remains a significant challenge because of the limited ability to perform biopsies in order to understand what aberrant mechanisms are occurring on a molecular level.

There are myriad of etiologies that can lead to hearing loss, including: genetic, infectious, noise trauma, and multifactorial disorders such as presbycusis. Clinicians often rely on an assortment of objective testing, including audiometry and vestibular testing, to guide diagnosis and treatment. While these tests provide a measure of function, they do not provide a molecular diagnosis and often do not correctly reflect the cellular site of lesion^2^. MiRNAs are 19–23 base pair single stranded RNA sequences that regulate post translational gene expression^3^. These molecules have been identified in all body fluids and are recognized for their promising role as a diagnostic and prognostic marker for neurodegenerative diseases such as Alzheimer’s and various cancers^4–8^. We recently demonstrated that miRNA profiling within the inner ear is a feasible methodology and can potentially offer insight into what is occurring on a cellular and molecular level in various inner ear pathologies^9^. In our search for specific hearing loss related biomarkers, we were able to demonstrate that various inner ear diseases, from Meniere’s disease to otosclerosis, express different and distinct miRNA profiles^9^. Similarly, recent investigations have also identified several key and distinct miRNAs within the venous blood in patients with sudden sensorineural hearing loss compared to healthy controls^10^. However, one of the challenges facing analysis of miRNA data from the inner ear is the immense and variable expression patterns across various diseases that may not be common to all cases.

Machine learning (ML) is a subdiscipline of artificial intelligence (AI) and borrows from multiple disciplines including mathematics, statistics, and computer science^11,12^. The field of ML is broadly concerned with two types of tasks: supervised and unsupervised learning. Supervised learning uses prior information on the outcome of interest (labeled data) with the goal of learning a function that, given data on the both the outcome and predictor variables, best approximates the relationship between the predictors and outcome. Supervised learning can be further subdivided into classification and regression depending on the nature of the outcome variable; the former being used when the outcome is categorical and the latter when the outcome is continuous. Conversely, unsupervised learning does not use labeled outputs, but rather seeks to learn and infer the underlying structure present within a set of data. An example unsupervised learning would the use of gene expression microarray data to identify molecular subtypes of a given disease, or otherwise groups/clusters of subjects with a similar gene expression profile. Simply put, ML methods are used by researchers to analyze large amounts of data to find patterns, and in doing so, better solve problems. With the ever-advancing nature of computing power, ML has gained significant popularity within the scientific community. While ML has been slow to assimilate into healthcare, we have seen ML applications ranging from diagnosing skin cancer^13^, diagnosing brain tumors through MRI^14^, diagnosing glaucoma^15^, optimizing drug therapies^16^, analyzing large genome sequencing^17,18^, to predicting various diseases and clinical outcomes^12,19–21^.

In the current study, we used ML to build disease specific algorithms to predict the presence of sensorineural hearing loss in different inner ear pathologies based on perilymph-derived miRNA expression profiles of the inner ear. Subsequently, we applied our algorithms to de-identified patient samples and established the presence and varying degree of sensorineural hearing loss through miRNA expression profile alone. This methodology offers a promising approach for inner diagnosis, prognosis, and monitoring for various neurotologic diseases. Likewise, using this approach we may be able to understand what may be occurring on a molecular level in inner ear disease specific states in a manner that was previously not possible.

## Results

**Figure 1:**
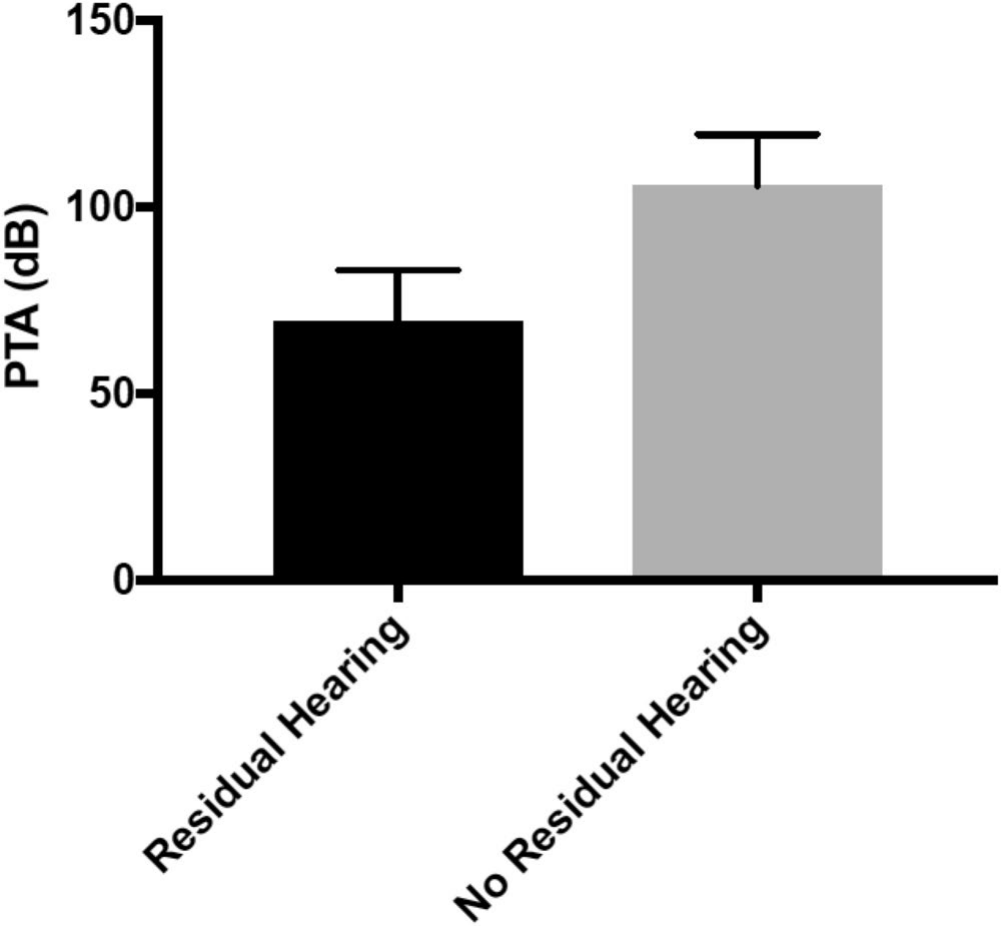
Average pure tone average (PTA) in decibels (dB) for cochlear implant patients with sensorineural hearing loss with and without residual hearing.

We collected perilymph from a total of sixteen patients. Four patients had otosclerosis and perilymph was collected while undergoing stapedectomy. These patients had a pure conductive hearing loss and served as controls since sampling perilymph from patients without an active ear disorder that requires a surgery is not possible at this point in time. Twelve patients underwent cochlear implantation in which perilymph was collected upon opening up the round window for electrode insertion. Seven out of twelve patients had profound SNHL and were classified as not having any residual hearing (PTA > 80 dB), with a mean PTA of 108.9 dB. A total of five out of twelve patients with severe SNHL were classified as having residual hearing (PTA < 80 dB), with a mean PTA of 69.4 dB (Fig. 1). As the use of ML to predict inner ear disease using miRNA signatures represents a novel application, we elected to build and test multiple ML models to ascertain if one was superior to others.

### Conductive Hearing Loss versus Sensorineural Hearing Loss

A ML model was constructed to differentiate between SNHL (patients undergoing cochlear implantation) and CHL (patients undergoing stapedectomy). Using a 70/30 split of the data into training/testing sets, both the decision forest and logistic regression ML models were able to differentiate between SNHL and CHL with 100% accuracy in the testing set. The decision jungle and neural network ML were able to differentiate between SNHL and CHL with 80% accuracy in the testing set. We cross validated the models using a leave-one-out cross approach, and all 4 models had a misclassification error of 6.25%. In order to understand how the model was built, we applied the permutation feature of importance to the ML models. The permutation feature of importance is a feature option within Azure ML software that allows the model to understand and evaluate the weighted importance of each data point, in this case miRNA, within the constructed model. The most heavily weighted miRNAs used to construct the ML model included: human miRNA 4767, miRNA 182 5p, miRNA 6754 5p, miRNA 6797 3p, miRNA 6806 3p, miRNA 6860, miRNA 4278, miRNA 3975, miRNA 4655, and miRNA 4732. The miRNAs were considered significant if they were used in the construction of two or more ML models. The miRNAs identified were compared to gene expression in the inner ear, and miRNA function and potential relationship to hearing loss was evaluated using Ingenuity Pathway Analysis (IPA) software. miRNA 4767, 6754 5p, 6797 3p, 6860, and 4732 had no significant known interactions identified. On the other hand, miRNA 182 5p, 6806 3p, 4278, 3975, and 4655 were identified as high probability or with proven interactions in previous microRNA – gene interaction experiments. Table 1 summarizes the key miRNAs with predicted downstream interactions identified that were significant in building ML model to differentiate SNHL from CHL.

**Table 1:** Critical miRNA identified and predicted downstream gene expression targets for sensorineural hearing loss vs conductive hearing loss.

miRNA 182	Entrez Gene Name	Location	Family	miRNA - mRNA Match
ADCY6	Adenylate Cyclase 6	Plasma Membrane	enzyme	Proven
IGF1R	Insulin Like Growth Factor 1 Receptor	Plasma Membrane	transmembrane receptor	Proven
MITF	Melanogenesis Associated Transcription Factor	Nucleus	transcription regulator	Proven
MTDH	Metadherin	Cytoplasm	transcription regulator	Proven
PPARA	Peroxisome Proliferator Activated Receptor Alpha	Nucleus	ligand-dependent nuclear receptor	Proven
RARG	Retinoic Acid Receptor Gamma	Nucleus	ligand-dependent nuclear receptor	Proven
TP53	Tumor Protein p53	Nucleus	transcription regulator	Proven
**miRNA 4278**				
ARCN1	archain 1	Cytoplasm	other	High Probability
CASC4	cancer susceptibility 4	Cytoplasm	other	High Probability
CD81	CD81 molecule	Plasma Membrane	other	High Probability
EIF4A1	eukaryotic translation initiation factor 4A1	Cytoplasm	translation regulator	High Probability
FOSB	FosB proto-oncogene, AP-1 transcription factor subunit	Nucleus	transcription regulator	High Probability
GDI1	GDP dissociation inhibitor 1	Cytoplasm	other	High Probability
HSP90B1	heat shock protein 90 beta family member 1	Cytoplasm	other	High Probability
KAT2B	lysine acetyltransferase 2B	Nucleus	transcription regulator	High Probability
NT5DC3	5′-nucleotidase domain containing 3	Other	other	High Probability
RHOC	ras homolog family member C	Plasma Membrane	enzyme	High Probability
SRSF2	serine and arginine rich splicing factor 2	Nucleus	transcription regulator	High Probability
STOM	stomatin	Plasma Membrane	other	High Probability
THRA	thyroid hormone receptor, alpha	Nucleus	ligand-dependent nuclear receptor	High Probability
**miRNA 3975**				
ACTG1	actin gamma 1	Cytoplasm	other	High Probability
BSG	basigin (Ok blood group)	Plasma Membrane	transporter	High Probability
DCTN5	dynactin subunit 5	Cytoplasm	other	High Probability
EPS15	epidermal growth factor receptor pathway substrate 15	Cytoplasm	other	High Probability
GJB2	gap junction protein beta 2	Plasma Membrane	transporter	High Probability
IFI6	interferon alpha inducible protein 6	Cytoplasm	other	High Probability
PPP1R1B	protein phosphatase 1 regulatory inhibitor subunit 1B	Cytoplasm	phosphatase	High Probability
SC5D	sterol-C5-desaturase	Cytoplasm	enzyme	High Probability
**miRNA 4655**				
CDC42	cell division cycle 42	Cytoplasm	enzyme	High Probability
FAM126A	family with sequence similarity 126 member A	Cytoplasm	other	High Probability
FOXO1	forkhead box O1	Nucleus	transcription regulator	High Probability
GPX1	glutathione peroxidase 1	Cytoplasm	enzyme	High Probability
NFIX	nuclear factor I X	Nucleus	transcription regulator	High Probability
SERPINF1	serpin family F member 1	Extracellular Space	other	High Probability
THRA	thyroid hormone receptor, alpha	Nucleus	ligand-dependent nuclear receptor	High Probability
UBE3A	ubiquitin protein ligase E3A	Nucleus	enzyme	High Probability
XRCC6	X-ray repair cross complementing 6	Nucleus	enzyme	High Probability

### Severity of sensorineural hearing loss

**Figure 2:**
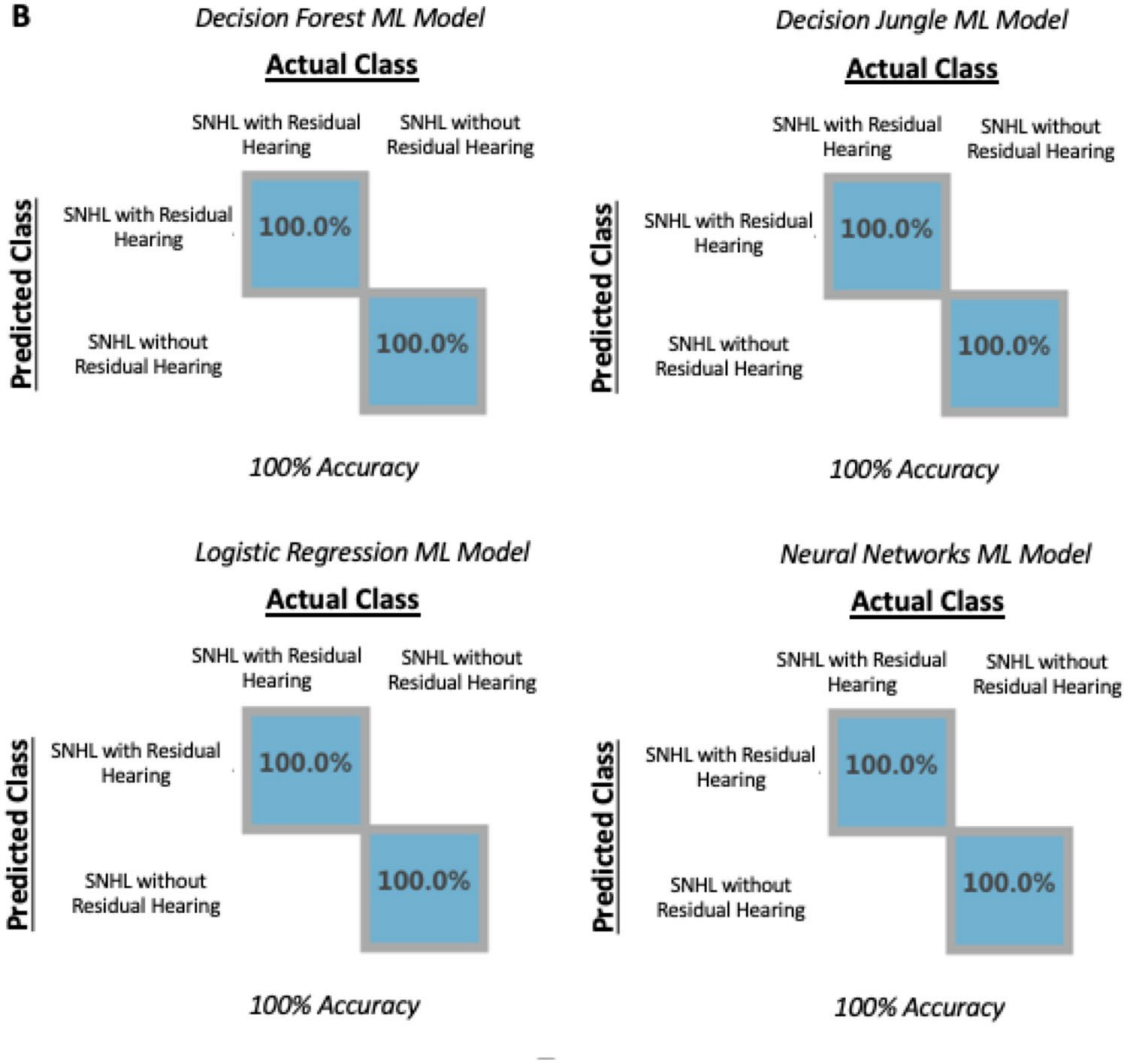
Representative evaluation and scoring maps for machine learning (ML) training models and testing set. X axis represents the actual diagnosis while the Y axis represents the predicted diagnostic class based on the testing set. (**A**) Decision forest and decision jungle ML model built to diagnose sensorineural hearing loss (SNHL) compared to conductive hearing loss (CHL) using a 70/30 split. Decision forest is able to diagnose with 100% accuracy while decision jungle is able to diagnose with 80% accuracy. (**B**) Decision forest, decision jungle, logistic regression, and neural networks ML models built to diagnose SNHL with and without residual hearing. All four ML models were able to diagnose with 100% accuracy.

A ML model was constructed to differentiate between cochlear implant candidates with different degrees of sensorineural hearing loss. Groups were categorized as either having residual hearing (cochlear implantation patients with PTA < 80 dB) or no residual hearing (cochlear implant patients with PTA > 80 dB). All four ML models, which included: decision forest, decision jungle, logistic regression, and neural networks, were able to differentiate between cochlear implant candidates with and without residual hearing with 100% accuracy (Fig. 2B). To cross validate these models, we used a leave-one-out cross validation approach. The misclassification error for each model is: decision forest, 0%; logistic regression, 8.33%; decision jungle, 25%; and neural network, 41.67%. In order to understand how the model was built, we applied the permutation feature of importance to the ML models. The most heavily weighted miRNAs used to construct the ML model included human miRNA 184, miRNA 660, miRNA let 7a 5p, miRNA 3142, and miRNA 335. The miRNAs were considered significant if they were used in the construction of two or more ML models. The miRNAs identified were compared to gene expression in the inner ear, and miRNA function and potential relationship degree of SNHL were evaluated using IPA software. Table 2 summarizes the key miRNA and predicted downstream targets which included miRNA 184, miRNA 660, and miRNA let 7a 5p. No significant known interactions were predicted using IPA software for miRNA 3142 and miRNA 335.

**Table 2:** Critical miRNA identified and predicted downstream gene expression targets for sensorineural hearing loss with and without residual hearing.

miRNA -184	Entrez Gene Name	Location	Family	miRNA - mRNA Match
AKT2	AKT serine/threonine kinase 2	Cytoplasm	Kinase	Proven
NFATC2	nuclear factor of activated T cells 2	Nucleus	transcription regulator	Proven
ADD1	adducin 1	Cytoplasm	Other	High Probability
AGRN	agrin	Plasma Membrane	Other	High Probability
APLN	apelin	Extracellular Space	Other	High Probability
BCL2L1	BCL2 like 1	Cytoplasm	Other	High Probability
CAMK2B	calcium/calmodulin dependent protein kinase II beta	Cytoplasm	kinase	High Probability
CDC25A	cell division cycle 25A	Nucleus	phosphatase	High Probability
CSF1	colony stimulating factor 1	Extracellular Space	cytokine	High Probability
CYCS	cytochrome c, somatic	Cytoplasm	transporter	High Probability
GRIN1	glutamate ionotropic receptor NMDA type subunit 1	Plasma Membrane	ion channel	High Probability
HTR1A	5-hydroxytryptamine receptor 1A	Plasma Membrane	G-protein coupled receptor	High Probability
ID1	inhibitor of DNA binding 1, HLH protein	Nucleus	transcription regulator	High Probability
IL15RA	interleukin 15 receptor subunit alpha	Plasma Membrane	transmembrane receptor	High Probability
IL7R	interleukin 7 receptor	Plasma Membrane	transmembrane receptor	High Probability
IQSEC. 3	IQ motif and Sec. 7 domain 3	Cytoplasm	other	High Probability
LASP1	LIM and SH3 protein 1	Cytoplasm	transporter	High Probability
LRRC8A	leucine rich repeat containing 8 VRAC subunit A	Plasma Membrane	ion channel	High Probability
LYNX1	Ly6/neurotoxin 1	Plasma Membrane	transporter	High Probability
NFATC2	nuclear factor of activated T cells 2	Nucleus	transcription regulator	High Probability
NFATC2IP	nuclear factor of activated T cells 2 interacting protein	Nucleus	other	High Probability
PACSIN1	protein kinase C and casein kinase substrate in neurons 1	Cytoplasm	kinase	High Probability
PDGFB	platelet derived growth factor subunit B	Extracellular Space	growth factor	High Probability
PGA5 (includes others)	pepsinogen 3, group I (pepsinogen A)	Extracellular Space	peptidase	High Probability
PIGQ	phosphatidylinositol glycan anchor biosynthesis class Q	Cytoplasm	enzyme	High Probability
PLPP3	phospholipid phosphatase 3	Plasma Membrane	phosphatase	High Probability
PLTP	phospholipid transfer protein	Extracellular Space	enzyme	High Probability
S100A7A	S100 calcium binding protein A7A	Cytoplasm	other	High Probability
SIRPA	signal regulatory protein alpha	Plasma Membrane	phosphatase	High Probability
SLA	Src like adaptor	Plasma Membrane	other	High Probability
SRC	SRC proto-oncogene, non-receptor tyrosine kinase	Cytoplasm	kinase	High Probability
TNK2	tyrosine kinase non receptor 2	Cytoplasm	kinase	High Probability
TSPEAR	thrombospondin type laminin G domain and EAR repeats	Extracellular Space	other	High Probability
VAMP1	vesicle associated membrane protein 1	Cytoplasm	transporter	High Probability
**miRNA-660**				
AOC3	Amine Oxidase, Copper Containing 3	Plasma Membrane	enzyme	High Probability
APOBEC3F	Apolipoprotein B MRNA Editing Enzyme Catalytic Subunit 3F	Nucleus	Deaminase	High Probability
CDH13	Cadherin 13	Plasma Membrane	other	High Probability
DNTT	DNA Nucleotidylexotransferase	Nucleus	DNA polymerase	High Probability
EXOC1	Exocyst Complex Component 1	Cytoplasm	Transporter	High Probability
FOLH1	Folate Hydrolase 1	Plasma Membrane	enzyme	High Probability
GRIN2B	Glutamate Ionotropic Receptor NMDA Type Subunit 2B	Plasma Membrane	transmembrane receptor	High Probability
HIF1A	Hypoxia Inducible Factor 1 Subunit Alpha	Nucleus	transcription regulator	High Probability
KCNJ2	Potassium Voltage-Gated Channel Subfamily J Member 2	plasma Membrane	ion channel	High Probability
NRCAM	Neuronal Cell Adhesion Molecule	Plasma Membrane	other	High Probability
QPRT	Quinolinate Phosphoribosyltransferase	Cytoplasm	enzyme	High Probability
SAA1	Serum Amyloid A1	Cytoskeleton	other	High Probability
VDAC1	Voltage Dependent Anion Channel 1	Plasma Membrane	ion channel	High Probability
**Let 7a 5p**				
FAM105A	family with sequence similarity 105 member A	Other	other	Proven
FAM96A	family with sequence similarity 96 member A	Extracellular Space	other	Proven
GRPEL2	GrpE like 2, mitochondrial	Cytoplasm	other	Proven
KCNJ16	potassium voltage-gated channel subfamily J member 16	Plasma Membrane	ion channel	Proven
MARS2	methionyl-tRNA synthetase 2, mitochondrial	Cytoplasm	enzyme	Proven
MIR4500	microRNA 4500	Cytoplasm	microRNA	Proven
SLC1A4	solute carrier family 1 member 4	Plasma Membrane	transporter	Proven
SLC38A1	solute carrier family 38 member 1	Plasma Membrane	transporter	Proven
SMOX	spermine oxidase	Cytoplasm	enzyme	Proven
SYPL1	synaptophysin like 1	Plasma Membrane	transporter	Proven
ADAMTS8	ADAM metallopeptidase with thrombospondin type 1 motif 8	Extracellular Space	peptidase	High Probability
AGBL2	ATP/GTP binding protein like 2	Cytoplasm	enzyme	High Probability
DSCR8	Down syndrome critical region 8 (non-protein coding)	Other	other	High Probability
FRMD4B	FERM domain containing 4B	Cytoplasm	other	High Probability
INTS6L	integrator complex subunit 6 like	Nucleus	other	High Probability
MIR4500	microRNA 4500	Cytoplasm	microRNA	High Probability
PQLC2	PQ loop repeat containing 2	Cytoplasm	transporter	High Probability
SDR42E1	short chain dehydrogenase/reductase family 42E, member 1	Other	enzyme	High Probability
SLC35D2	solute carrier family 35 member D2	Cytoplasm	transporter	High Probability
TMEM211	transmembrane protein 211	Other	other	High Probability
TTLL4	tubulin tyrosine ligase like 4	Cytoplasm	enzyme	High Probability

A regression ML model was constructed using pure tone average (PTA) in decibels to further study the relationship between miRNA and severity of SNHL. The root mean squared error for our best model was 21.26 dB, allowing us to predict hearing loss with an expected error in the 21 dB range. We observed a decrease in sensitivity of the model when comparing directly with PTA as a continuous variable, compared to categorical characterization using 80 dB as a cut off. The decreased accuracy is consistent with the heterogenous nature of hearing loss within our patient cohort.

## Discussion

MiRNAs were initially discovered in 1993 and have since been shown to play an essential role in post translational regulation of gene expression through messenger RNA (mRNA) degradation and splicing^3,22^. Because miRNA play a critical role in cell gene expression, miRNA activity has been increasingly recognized as a critical component to many disease states making them a promising biomarker^5,23^. Interestingly, miRNA profiles have shown unique expression patterns to different diseases states, from heart failure^24^ to individual cancers^7^ (colon^25–27^, ovarian^28^, and clear cell carcinoma^29^), neurodegenerative diseases^4^, different cell types^30^, and play an integral role embryonic development^8^. Pertinent to hearing loss, miRNA have shown to be a promising biomarker and diagnostic marker for otherwise difficult to diagnose neurodegenerative diseases such as Alzheimer’s and Parkinsons^4,5,31^. Additionally, miRNA have shown to play a critical role in inner ear development, demonstrate tissue and site specific expression, and may exhibit expression patterns specific to SNHL^10,32–34^. Taken together, miRNA expression profiling may serve as a promising diagnostic and prognostic tool for the inner ear. We sought to see if ML could differentiate between distinct inner ear miRNA expression profiles specific to different active inner ear disease states.

To date, we have no biopsy equivalent for inner ear disease and have no objective insight into what is occurring on a molecular level in patients with active inner ear disease. The knowledge we have assimilated to date is based on objective testing and the study of various otologic and neurotologic pathologies using temporal bone pathology and animal models. Here, we have shown that various inner ear diseases may demonstrate specific and unique miRNA expression profiles in the very simple model of presence or absence of sensorineural loss, and a model differentiating varying severity of sensorineural hearing loss. Utilizing the unique expression profiles, we can use machine learning to build algorithms to differentiate between different degrees of sensorineural hearing loss with high accuracy, opening the door to further refining the application of this methodology.

Artificial intelligence (AI) and its subdiscipline machine learning (ML) have seamlessly integrated themselves into our modern-day culture, but they have been slow to assimilate into health care. ML enables one to analyze large amounts of data, understand pattern recognition, and make predictions using what it has learned. ML borrows from many subdisciplines including statistics and computer science. However while statistics primarily focuses on inferences related to causation and examines how a system of components relate to one another, ML is novel in that it makes predictions based on large sets of data and experiences^35^. We can supply an un-analyzed perilymph miRNA profile into our ML algorithms and subsequently make accurate predictions to discern between different degrees of sensorineural hearing loss. By analyzing the function of miRNAs identified by this process we may be able to indirectly identify the molecular pathways involved in different inner ear diseases.

ML is optimally used when applied to large and complex datasets, such as gene expression profiles, where it can analyze various patterns to make predications without being specifically programmed to do so. Using the permutation feature of importance we can gain significant insight into how the different decision algorithms are built and how certain factors are weighted (Tables 1 and 2). ML and permutation feature of importance also offers a novel way to analyze miRNA that may be playing a critical role in various inner ear pathologies. For example, looking at SNHL vs CHL, we inputted the critical miRNA identified (4278, 3975, 4655) into the IPA software, and isolated downstream targets that have been experimentally proven. We then analyzed downstream targets that overlap with two or more of the critical miRNA identified. Of interest we identified KCNJ10, HCN, and Otoferlin. All three genes have been experimentally proven with the individual miRNAs and shown to play critical roles in SNHL on a molecular level. KCNJ10 has been shown within the stria vascularis to have a critical role in generating endocochlear potentials^36^. Hyperpolarized-activated cyclic nucleotide cation (HCN) channels have been shown to play an essential role within the spiral ganglia and propagating post synaptic potentials^37^. Otoferlin has been shown to play a critical role in Ca2+ evoked vesicular exocytosis within inner hair cells^38^. Similarly, miRNA Let 7 family was critical in differentiating CI patients with and without residual hearing. Investigators have experimentally shown how Let 7 miRNA family plays a critical role in RAS signaling^39^, and along similar lines RAS/MAPK pathway is crucial in inner hair survival following noise induced hearing loss^40^. The unique miRNA profiles identified through ML can offer significant understanding to aberrant disease mechanisms on a molecular level and also potentially identify site specific pathology.

Furthermore, using SNHL with and without residual as a categorical differentiation we are able to construct ML models with nearly 100% accuracy. However, when analyzing PTA as a continuous variable we lose some accuracy with a root mean squared error of 21.26 dB, allowing us to predict +/−21 dB. While these results do raise some inconsistency issues using miRNAs as a direct measure to varying degrees of SNHL, they are not surprising given the heterogeneity of hearing loss within our patient cohorts. PTA is an average hearing loss across multiple frequencies. While patients may have similar PTAs, they can have significantly different patterns and frequencies affected. Secondly patients have different underlying disease mechanisms leading to hearing loss, such as presbycusis versus early onset genetic hearing loss. Taking both these factors into account, the decreased accuracy using PTA as a continuous variable is not surprising. As we continue to grow our patient perilymph database and homogenize our SNHL patients with similar patterns and frequencies, we would expect an improvement within our regression ML model.

Despite the increasing recognition of the unique role miRNAs play in development and various disease states, identifying and validating miRNA target genes has been a great challenge^41,42^. Computational analysis on miRNAs and regulation of gene expression are based on aligning and predicting 5′ miRNA and 3′ complimentary known mRNA sequences. While this methodology is the mainstay of miRNA discovery and gene expression analysis, it does have its limitations^42,43^. As we continue to grow our miRNA perilymph database, key and critical miRNA identified will need to be validated through miRNA/mRNA target validation, co expression, and ultimately their regulatory effect on gene expression through luciferase and other validated expression assays^41^. Validation experiments will be critical in moving forward, particularly as the key miRNA unique to different inner ear disease states are pursued as potential drug therapy targets.

While the ML methodologies employed herein offer an exciting prospect, we must acknowledge several limitations. Definitive conclusions are limited by our small sample size of 16 patients. As we continue to grow our database and include a wider range of pathologies, we do not expect to maintain 100% accuracy in our predictions; however, we do expect the sensitivity of our algorithms to improve. ML algorithms are adaptive, therefore, as we continue to recruit additional patients, the ML algorithms will continue to learn from these new “experiences” and adapt its output accordingly. However, our first goal was to demonstrate that inner ear miRNA expression profiling may be unique to disease specific inner ear pathologies. Using ML, we were able to successfully delineate these unique miRNA expression profiles and use them to predict ongoing inner ear pathologies in patients in real time, a methodology not previously possible on a molecular level.

Finally, one of the major limitations of ML is the “black box” that are the predictive algorithms. We can control the data that goes in and the desired predictions we wish to build, but we have limited insight into the exact mechanics behind each algorithm^44^. One of the shortcomings of ML as compared to traditional statistical methodologies is that we cannot put any significantly meaningful inferences behind how the model is built. There are no confidence intervals or odds ratios equivalent for ML. The only meaningful insight we have into the algorithm is using the different analytical features such as permutation feature of importance to assess the contribution of each miRNA to the constructed model. While we can understand which limited number of the miRNA being used and at what weighted fraction, unfortunately we do not know if its upregulation or downregulation of a pathway for each specific miRNA. There are many ongoing efforts to understand and unlock this “black box” but unfortunately we still have yet to find a definitive solution^45^.

## Conclusions

MiRNAs’ unique role and expression profiles are well recognized to play an integral role in different disease states from cancer to neurodegenerative states making them an exciting biomarker. MiRNAs are well established to play a critical role in inner ear development and some preliminary investigations have shown its potential role in SNHL. In this study, we demonstrate using ML we can delineate the miRNA expression profile unique to different inner ear pathologies. This methodology not only provides an understanding of inner ear pathology on a molecular level, but may also offer a novel method to diagnose and prognose patients with active inner ear disease. Similar to how a patient undergoes a lumbar puncture to collect CSF for meningitis, one could theoretically undergo a “round window tap” to diagnose, prognose, and monitor therapeutic interventions for Meniere’s disease or SNHL. While our methodology may offer an exciting prospect for stratification of patients with inner ear disease, multiple safety and validation studies will need to be performed. Additional patient recruitment will be needed to continue to grow our perilymph miRNA database to improve the predictive algorithms and its potential ability to differentiate various inner ear pathologies.

## Methods

Human perilymph sampling was approved by the University of Kansas Human Studies Committee and Institutional Review Board (IRB). All experiments performed were in accordance with relevant guidelines and regulations approved by the University of Kansas IRB. Patients were recruited if they were undergoing a surgical procedure in which the inner ear was opened, and informed consent was obtained prior to perilymph collection. Procedures in which patients were recruited included patients undergoing stapedectomy for otosclerosis or cochlear implantation for sensorineural hearing loss (SNHL). All patients received standard of care and underwent standard surgical treatment for either stapedectomy or cochlear implantation. Only difference is when patient’s inner ear was opened for their indicated procedure, a small sterile glass capillary tube was used to collect approximately 2–5 µL volume of perilymph^9^.

### Sampling for stapedectomy

The skin of the external auditory canal was injected with 0.5 ml of 1% lidocaine + 1:100,000 epinephrine. Using a round knife, a cut was made in the skin of the external canal and the dependent middle flap was carefully elevated medially revealing the middle ear structures. Using the Omniguide™ CO2 laser with a power setting a four watts, 0.1 second single bursts, the stapes superstructures were removed. Using the laser, a rosette fenestration was made in the stapes footplate. Upon making the fenestration, perilymph could be seen coming out laterally from the vestibula. We then removed excess perilymph with a sterile glass capillary tube. After successful collection of perilymph fluid, the stapes footplate fenestration was enlarged to accommodate the stapes prosthesis. The prosthesis was then hooked around the incus and the surgery was completed in a standard fashion.

### Sampling for cochlear implantation

Through a post auricular incision, a mastoidectomy and facial recess exposure of the round window were completed in a standard fashion. The wound was irrigated with antibiotic solution. The oval and round windows were identified, and the bony overhang of the round window niche was removed with a 1 mm micro drill. Using an angled pick, the round window was opened. At this point there was free flow of excess perilymph out of the cochlea which was sampled using a sterile glass capillary tube. The implant electrode was then inserted into the cochlea.

### microRNA analysis

The perilymph was collected as described above. Total RNA was extracted with Trizol reagent (Thermofisher, cat #15596018) and purified by centrifuging with phase lock heavy gel (Tiagen, cat # WMS-2302830). RNA was analysed using The Agilent RNA6000 Pico kit using an Agilent Bioanalyzer 2100 yielding on average 0.5–2 ng of total RNA per sample. Samples were processed and analysed with an Affymetrix miRNA 4.0 array to determine the presence of micro RNAs. The Affymetrix miRNA 4.0 array interrogates all miRNA sequences listed in miRBase Release 20; interrogating 30,434 mature miRNAs from 203 organisms of which 2,578 are from humans. The arrays were background corrected, normalized and gene-level summarized using the Robust Multichip Average (RMA) algorithm^9^. This normalization step makes inferences on miRNA expression across conditions possible. In order to ascertain which miRNAs were significantly expressed in each array, for each miRNA probe in the array, a detection p-value was computed based on a Wilcoxon Rank-Sum test of the miRNA probe set signals compared to the distribution of a GC matched background signal comprising of anti-genomic probes in the same array. The detection p-values were adjusted for multiple hypothesis testing (FDR) using the Benjamini and Hochberg method. These analyses were performed using Affymetrix Expression Console Software. miRNAs with a normalized log2 signal intensity ≥7 and an adjusted detection p-value (FDR) ≤ 0.05 were considered significantly expressed in the assay and were considered in downstream ML model development.

### Data analysis

#### Patient selection and audiometry

All patients underwent pure tone audiometry prior to their respective procedures. Air and bone conduction thresholds were determined, and a CT scan of the temporal bone was performed for all patients with diagnosis of otosclerosis. Only patients with conductive hearing loss (CHL) and radiologically confirmed otosclerosis were included in the CHL control group (n = 4). For patients that met audiologic criteria for cochlear implantation, the frequency pure tone average (PTA) was determined. A PTA of 70 dB was used as a limit identifying patients with residual hearing (PTA < 80 dB) (n = 5) and no residual hearing (PTA > 80 dB) (n = 7) (Fig. 1).

#### Machine learning analysis

**Figure 3:**
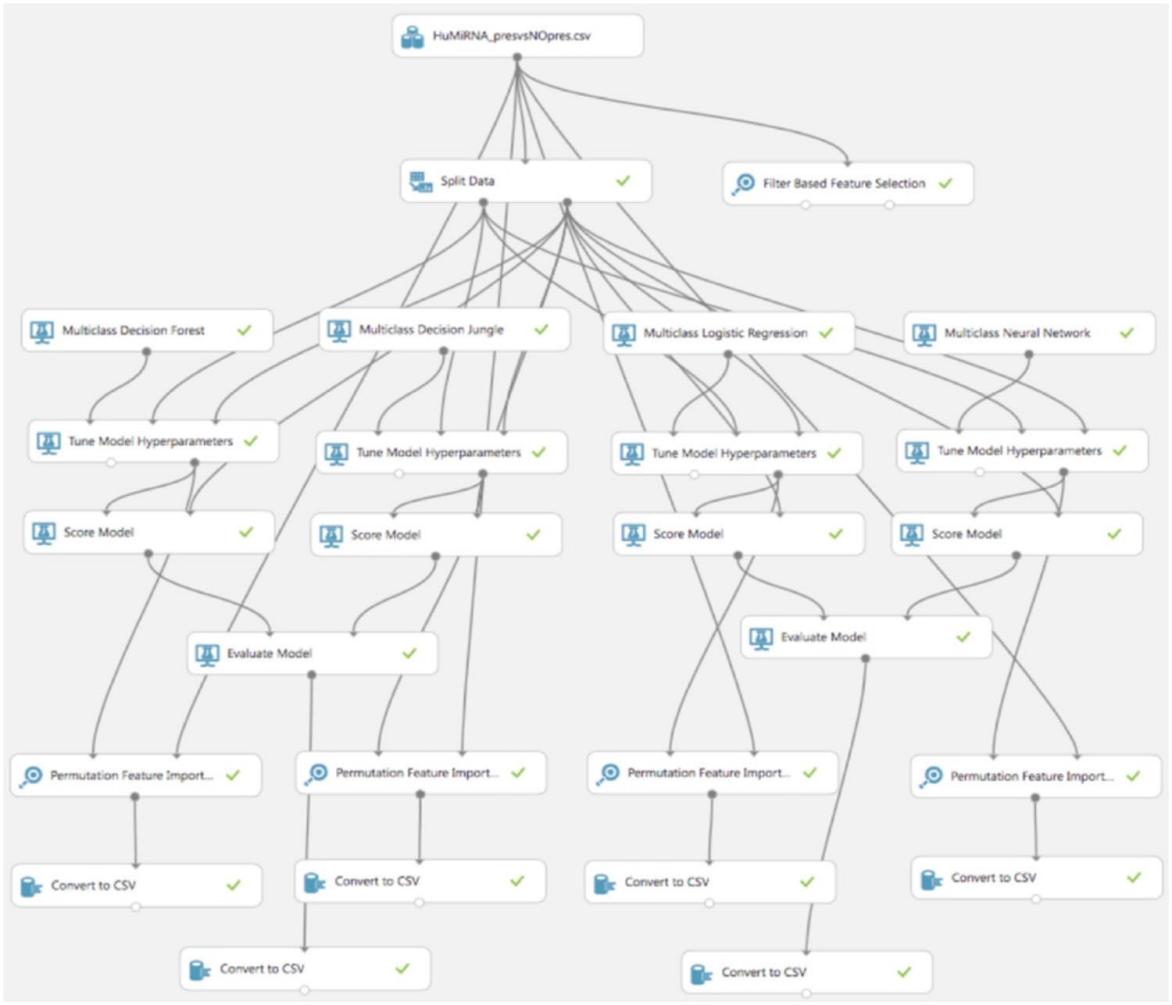
Representative machine learning (ML) experiment mapped for sensorineural hearing loss with and without residual hearing using Azure Machine Learning Studio (Microsoft Corporation).

In order to analyse the miRNA dataset unique to various inner ear pathologies, we constructed a supervised machine learning classification model using the opensource Azure Machine Learning Studio (Microsoft Corporation). Data was uploaded form the Affymetrix miRNA 4.0 array, and formatted for Azure ML. The log2 transformed signal intensities were used to construct the ML models. The data was randomly split 70/30 into training and testing sets. ML models were built using the training data and subsequently tested on the remaining 30% of data comprising the testing set. We considered multiple multiclass decision models, including: multiclass decision forest (minimum samples per lead node 1; number of random splits 128; maximum depth of decision tree 32; number of decision trees 8), multiclass decision jungle (number of optimization steps per decision DAG layer 2048; maximum width of decision DAGs 128; maximum depth of the decision DAGs 32; number of decision DAGs 8), multiclass logistic regression, and multiclass neural network. Boostrap aggregation was built into model generation for each of the fitted models. “Tune model hyperparameters”, an option within Azure ML software, was used to apply an entire grid wide sweep and determine the optimum parameters settings. After the model was built and tuned, it was then scored and evaluated using the testing set data (Figs 2 and 3). A leave-one-out cross validation approach was subsequently used to better evaluate each model’s performance. To represent the results of the cross validation, the leave-one-out misclassification error rate was determined for each model as the ratio of the number of misclassified samples to the total number of samples. A permutation-based approach was applied to assess feature importance within each multiclass trained decision model in order to analyse which miRNAs had the most influence, along with their corresponding weight in construction of the respective trained model. The metric used in the permutation feature analysis was accuracy, and permutation feature importance scores are determined as: permutation feature importance score = base model accuracy − model accuracy after shuffling a given feature. Thus, a high permutation feature importance score is indicative of a feature with a large influence on the model’s accuracy. We compared the ability of the models to distinguish between patients with CHL and SNHL (stapedectomy vs. cochlear implant patients) and within the cochlear implant group evaluated the ability of the models to distinguish between patients with and without residual hearing.

#### Evaluation of permutation features

Data analysis was performed along two fronts to understand pathogenesis and look for patterns of expression unique to different disease types. miRNAs identified as significantly influencing the model development were identified. Using Ingenuity Pathway Analysis (IPA) software (Qiagen Bioinformatics) these miRNAs were analysed alongside a human inner ear cDNA library. Known and highly predicted miRNA cochlear mRNA interactions were identified and mapped out to delineate and understand the different regulatory pathways.
